# Emerging roles and mechanisms of lncRNAs in fruit and vegetables

**DOI:** 10.1093/hr/uhae046

**Published:** 2024-02-23

**Authors:** Xiuming Zhao, Fujun Li, Maratab Ali, Xiaoan Li, Xiaodong Fu, Xinhua Zhang

**Affiliations:** College of Agricultural Engineering and Food Science, Shandong University of Technology, Zibo, 255000, Shandong, China; College of Agricultural Engineering and Food Science, Shandong University of Technology, Zibo, 255000, Shandong, China; College of Agricultural Engineering and Food Science, Shandong University of Technology, Zibo, 255000, Shandong, China; College of Agricultural Engineering and Food Science, Shandong University of Technology, Zibo, 255000, Shandong, China; College of Agricultural Engineering and Food Science, Shandong University of Technology, Zibo, 255000, Shandong, China; College of Agricultural Engineering and Food Science, Shandong University of Technology, Zibo, 255000, Shandong, China

## Abstract

With the development of genome sequencing technologies, many long non-coding RNAs (lncRNAs) have been identified in fruit and vegetables. lncRNAs are primarily transcribed and spliced by RNA polymerase II (Pol II) or plant-specific Pol IV/V, and exhibit limited evolutionary conservation. lncRNAs intricately regulate various aspects of fruit and vegetables, including pigment accumulation, reproductive tissue development, fruit ripening, and responses to biotic and abiotic stresses, through diverse mechanisms such as gene expression modulation, interaction with hormones and transcription factors, microRNA regulation, and involvement in alternative splicing. This review presents a comprehensive overview of lncRNA classification, basic characteristics, and, most importantly, recent advances in understanding their functions and regulatory mechanisms.

## Introduction

Fruit and vegetables are essential for a healthy diet as they provide vital nutrients. At the molecular level, the growth, development, and stress response of these plants are intricately regulated, influencing characteristics such as color, flavor, and texture [1]. According to the central dogma of molecular biology, genetic information is transcribed from DNA to RNA and then translated into proteins [2]. Recent genome-wide and transcriptome analyses have revealed that more than 50% of the *Arabidopsis* genome is transcribed. However, only about 1.5% of these transcripts undergo translation, and a significant portion are non-coding RNAs (ncRNAs) that do not code for proteins [3]. Previously considered as by-products of genomic transcription, ncRNAs have gained attention in the fields of epigenetics and other scientific disciplines. Extensive research has been conducted to explore the involvement of ncRNAs in the growth, development, and stress response of eukaryotes, providing a better understanding of their functional characteristics and regulatory mechanisms [4]. Long non-coding RNAs (lncRNAs) are a type of ncRNAs that are longer than 200 nucleotides (nt). Most lncRNAs are shorter in length and simpler in structure than protein-coding transcripts [5]. Some lncRNAs may contain open reading frames (ORFs), that could potentially encode short peptides of fewer than 100 amino acids, although the exact functions of these peptides remain enigmatic [6]. The first lncRNA, ENOD40, was discovered in 1993 [7], and research in this field has advanced significantly with progress in science and technology, including next-generation sequencing (NGS), microarray, and comparative genomics. Recent studies have uncovered various roles of lncRNAs in regulating the expression of protein-coding genes (PGs) at both transcriptional and post-transcriptional levels, thereby influencing the growth, development, and stress responses in fruit and vegetables. For instance, ACoS-AS1 is involved in fruit coloration [8], fruit ripening-related long intergenic RNA (FRILAIR) affects fruit maturation [9], and lncRNA33732 is associated with fruit resistance [10]. In the following sections, we summarize recent knowledge of the formation, functional characteristics, and regulatory mechanisms of lncRNAs in fruit and vegetables. Furthermore, we analyze the current state of studies on the roles and mechanisms by which lncRNAs mediate growth, development, and stress responses in fruit and vegetables, and explore the potential applications of these mechanisms in fruit and vegetable biotechnology and genetic breeding. By analyzing the latest research progress on lncRNAs in fruit and vegetables, our aim is to provide valuable references for further research and practical application in this field.

## Formation and classification of lncRNAs

lncRNAs play a vital role within plant regulatory networks. They are primarily transcribed and spliced by RNA polymerase II (Pol II) and exhibit limited evolutionary conservation. Most lncRNAs have 5′ caps and polyadenylation at the 3′ end, although some lack poly-A tails [11]. Notably, in plants, a minority of lncRNAs without poly-A tails are transcribed and spliced by plant-specific Pol IV/V. These particular lncRNAs exhibit lower expression levels and are characterized by high instability. LncRNAs produced through Pol IV and Pol V transcription play a crucial role in RNA-driven DNA methylation (RdDM), a complex and unique regulatory mechanism that contributes to plant genome stability [4].

**Figure 1:**
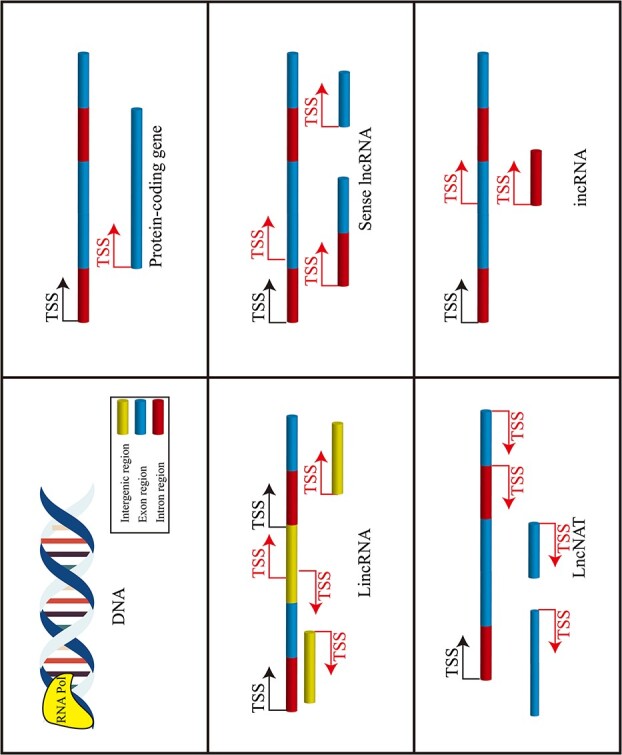
Biogenesis of lncRNAs. Four types of lncRNAs: intergenic lncRNAs (lincRNAs), sense lncRNAs, long non-coding antisense transcripts (lncNATs), and intronic lncRNAs (incRNAs). TSS, transcription start site. Yellow, blue, and red represent intergenic, exon, and intron regions, respectively. Black arrows denote the transcription direction and start site of the coding genes, whereas the red arrows represent the transcription direction and start site of the lncRNAs.

Based on their transcription direction and position relative to PGs, lncRNAs are classified into four types: intergenic lncRNAs (lincRNAs), long non-coding antisense transcripts (lncNATs), sense lncRNAs, and intronic lncRNAs (incRNAs) (Fig. 1). lincRNAs are mainly transcribed from regions located between two coding genes, while lncNATs originate from the 3′ end of coding genes and may overlap with coding gene exons. Conversely, sense lncRNAs are transcribed from the 5′ end of the genome [12]. lincRNAs predominate, followed by lncNATs. Sense lncRNAs, however, make up only a small portion (Table 1).

**Table 1:** Numbers of various types of lncRNAs in fruit and vegetables.

Crop	lncRNA	lincRNA	lncNAT	incRNA	Sense lncRNA	Library composition	Reference
*Actinidia chinensis*	27 402	17 344	2861	7197	0	Fruit maturity, postharvest ripening and soaked with ABA (softening stage)	[13]
	7051	6009	169	881	597	Fruit (beginning of cell division, onset of fruit maturation and onset of postharvest ripening)	[14]
	14 845	10 238	1874	75	1434	Leaf (0, 2, and 14 days post-inoculation with *Pseudomonas syringae* pv. *actinidiae*)	[15]
*Capsicum annuum*	2505	2389	21	95	0	Fruit (30, 40, and 50 days) and 06 J19-1-1-1-2 (30 days) after anthesis	[16]
	11 999	10 136	964	150	749	Fruit (40 and 60 days after anthesis)	[17]
*Capsicum chinense* Jacq. (Bhut Jolokia)	20 563	16 894	1396	717	1556	Fruit (flowering, mature green stage, and red ripe stage)	[18]
*Citrus limon*	11 814	0	0	0	0	Leaf, bud, fruit, and peel	[19]
*Cucumis melo*	3857	3307	550	0	0	Fruit (18, 20, 22 and 36 days after anthesis)	[20]
*Fragaria vesca*	25 613	11 959	13 398	256	0	Fruit (green achenes, yellow achenes, and brown achenes)	[9]
*Hippophae rhamnoides* L.	9008	6750	1052	1206	0	Fruit (46, 63, and 76 days post-anthesis)	[21]
	3428	2498	593	337	0	Red and yellow fruit	[22]
*Malus domestica*	9440	6976	308	731	1425	Phloem, leaves, flowers, and fruit	[23]
	5297	3441	439	352	1065	Postharvest bagged apples in light condition	[24]
	52 919	41 737	8764	0	2418	fruit (27, 84, 136, and 165 days after anthesis)	[25]
*Prunus persica*	1500	947	260	34	259	Fruit (30, 49, and 65 days after full blooming)	[26]
*Punica granatum*	3174	2420	400	240	114	Fruit (non-cracking, cracking, and non-cracking under bagging) late ripening	[27]
*Pyrus pyrifolia*	3332	2671	661	0	0	Ripening fruit (40 ± 2, 25 ± 1, and 4 ± 1°C)	[28]
*Solanum lycopersicum*	1411	1059	352	0	0	Fruit (72 h at 10 or 0°C)	[29]
	79 322	70 635	8085	0	602	Leaf, seeds, peel, seed, root, flesh, embryo, anther, buds, hypocotyls	[30]
	17 674	10 212	6232	1230	0	Fruit (5, 15, 35, 40, and 45 days after pollination)	[31]
	1834	1561	95	161	17	Fruit (37, 42, 46, 51, and 56 days post-anthesis)	[32]
	1845	1572	97	155	21	*rin* fruit (37, 42, 46, 51, and 57 days post-anthesis)	
	1044	859	165	9	11	*Solanum pennellii* and *S. lycopersicum* L. under salt stress for 12 h	[33]
*Vaccinium corymbosum* L.	25 036	12 306	3855	6372	0	Pad, cup, pink and blue fruit	[34]
*Vitis vinifera* L.	24 726	13 731	1868	9127	0	Berries (hard green, starting to soften and not quite ripe)	[35]

**Figure 2:**
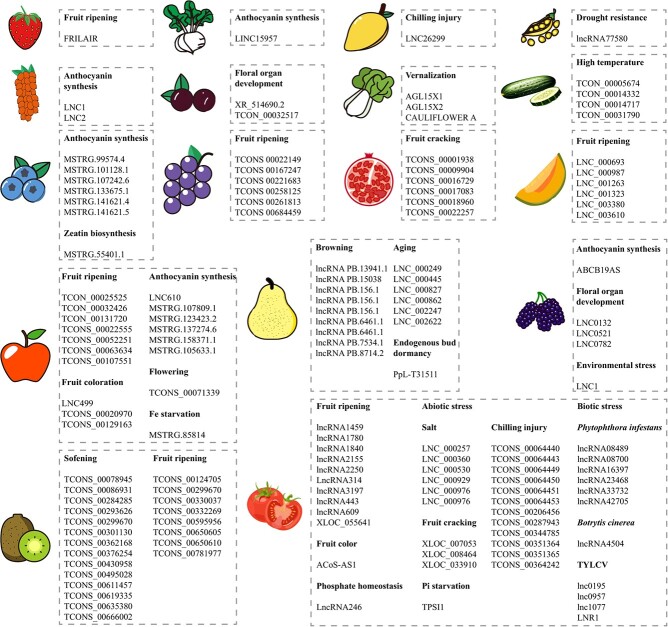
Functional diversity of lncRNA members in fruit and vegetables. The first row shows strawberries, radishes, mangoes, and peas; the second row shows sea buckthorn, plum, cabbage, and cucumber; the third row shows blueberries, grapes, pomegranates, and melons; the fourth row shows apple, pear, and mulberry; and the fifth row shows kiwi and tomato.

Numerous lncRNAs have been identified in various fruit and vegetables, exhibiting diverse characteristics and playing crucial roles in fruit development and regulation (Fig. 2).

## Basic characteristics of lncRNAs

lncRNAs found in fruit and vegetables are similar to mammalian lncRNAs in terms of sequence characteristics, expression patterns, and structural functions [36]. However, compared with the well-established research on mammalian lncRNAs, the study of lncRNA in fruit and vegetables is still in its early stages, particularly with regard to characteristic analysis, functional exploration, and mechanism analysis. For example, while research on mammalian lncRNAs has primarily focused on regulatory structural motifs, the analysis of lncRNA structural characteristics in fruit and vegetables is beginning to explore the secondary and tertiary structure levels [37]. Consequently, we have elucidated the attributes of current fruit and vegetable lncRNAs, including their abundance and size, structure and localization, as well as evolution and decay.

### Abundance and size

The transcription start sites and expression levels of lncRNAs often correlate with their nearest PG. lncRNAs frequently exert regulatory effects on neighboring genes, either in *cis* or *trans* mode, playing crucial roles in plant growth and development [4]. In contrast to PGs, lncRNAs tend to exhibit lower complexity in alternative splicing (AS), shorter lengths, and fewer exons and ORFs [13, 35, 38]. Notably, lncRNAs identified in *Fragaria vesca* mainly consist of lincRNAs and exonic lncNATs. Most lncRNAs are shorter than 5000 bp, with a few intronic lncNATs and incRNAs being under 2500 bp in length [9]. Antisense lncRNAs in kiwifruit are longer than other types of lncRNAs, while incRNAs are generally shorter. A common feature of these lncRNAs is that ~80% of lncNATs are multi-exon transcripts, whereas 80% of lincRNAs and incRNAs have only one exon [14]. Due to their widespread low expression specificity, lncRNAs exhibit greater tissue-specific and spatiotemporal expression patterns compared with PGs [6, 22]. For instance, in strawberry, numerous lncRNAs show specific expression during particular developmental stages [9]. In lemon tissues, including buds, fruit, peels, and leaves, differentially expressed lncRNAs (DELs) were observed, with 48, 424, 65, and 11 DELs identified in each tissue, respectively [19]. The number of DELs during different developmental stages of sea buckthorn fruit was reported to be much lower than that of mRNAs [21]. In addition, lncRNAs also exhibit divergent characteristics. lncNATs have the highest sequence conservation among lncRNAs, while lincRNAs show the highest number of differentially expressed transcripts in response to stress [6, 39]. The expression of lncNATs generally correlates positively with that of their adjacent homologous PGs, regardless of the transcriptional direction between lncNATs and their neighboring genes. Such a correlation is crucial for the expression of the adjacent PGs [40]. For example, 1059 of the 1411 lncRNAs responsive to cold stress in tomatoes were lincRNAs [29].

### Structure and localization

It is well known that lncRNAs can form complicated secondary and tertiary structures that are still not fully understood. These structures play a crucial role in the interaction between lncRNAs and other molecules, such as RNA, DNA, and proteins. In the complex regulatory network of plants, the expression changes of lncRNAs are extensively utilized to regulate gene expression and coordinate phenotypic modifications. Fabbri *et al*. [41] have identified two types of functional recognition sites for lncRNAs: interaction modules that physically interact with RNA-binding proteins through base complementarity, and structural modules located within the secondary and tertiary structures that facilitate interactions. Most lncRNAs contain both of these functional elements.

In general, lncRNA distribution across chromosomes is relatively even [24, 42]. However, there are exceptions to this characteristic in certain species. For example, in sea buckthorn [21], apple [27], and tomato [29] fruit, the distribution of lncRNAs in chromosomes is non-uniform. In apples, lincRNAs exhibit a dense distribution on chromosomes, while lncRNAs show the opposite pattern. Sense lncRNAs are mainly found at both ends of the chromosome, whereas lncNATs are primarily concentrated at one end of the chromosome [23]. lncRNAs exhibit significant sequence diversity but demonstrate strong conservation in their genomic position [26, 42]. Unlike mRNAs, which need transport to the cytoplasm for translation, lncRNAs can perform their functions within the nucleus or be transported to other cellular organelles following processing and modifications. The specific mechanisms and expression patterns of lncRNAs within regulatory networks vary significantly based on their subcellular localization. The distribution of lncRNAs between the nucleus and cytoplasm is unequal, with fewer lncRNAs in the cytoplasm. However, lncRNAs in the cytoplasm are more stable compared with those in the nucleus. The instability of nuclear lncRNAs arises from their turnover, similar to transcription factors (TFs) in gene regulation. This turnover leads to transcriptional changes in response to environmental stimuli [43].

### Evolution and decay

Compared with protein-encoding transcripts, lncRNAs are characterized by lower synthesis efficiency, faster metabolism and evolution rates, and decay patterns similar to those of mRNAs [12]. The emergence or decline of lncRNAs in biological systems can be attributed to genomic structural variations, with transposons being a key factor in these variations [44]. Transposons are a special class of DNA sequences that can appear at various genomic locations through transcription or reverse transcription under the action of endonucleases. The presence of transposons significantly contributes to genome formation and evolution. In strawberries, a total of 14 552 lncRNAs have been identified, with 59.2% derived from transposon elements [45].

## Role of lncRNAs in the growth, development, and stress response of fruit and vegetables

### Role of lncRNAs in the growth and development of fruit and vegetables

The significant role of lncRNAs in plant growth and development has been extensively described in numerous reviews, encompassing plant growth differentiation, photomorphogenesis, leaf morphology, and crop yield. Currently, extensive deep sequencing studies have identified a plethora of lncRNAs across various developmental stages and tissues in fruit and vegetables. These lncRNAs play crucial roles in diverse metabolic pathways throughout the growth and development of fruit and vegetables. In this paper we primarily focus on the intricate metabolic network regulated by lncRNAs during the growth and development of fruit and vegetables, spanning topics such as pigment accumulation, the development of reproductive tissue, and fruit ripening.

#### Pigment accumulation

The accumulation of pigment and its resulting changes in color are crucial characteristics in the ripening and development of fruit and vegetables. These pigments serve as bioactive compounds that reflect the unique nutritional and health attributes of these horticultural products. Furthermore, pigment accumulation is mostly influenced by the ripening stages. Monitoring the changes in pigment content serves as a significant indicator of fruit and vegetable development, profoundly influencing post-harvest storage and management [46]. Previous studies have shown that the lncNAT (*ACoS-AS1*) gene *trans*-splices the *PSY1* gene, which codes for phytoene synthase (PSY), resulting in the loss of PSY1 function. This genetic alteration subsequently results in yellow coloration in tomatoes [8]. In two grape varieties, a total of 25 699 lncRNAs were found in three stages of development. These lncRNAs support a number of functions during grape development, including photosynthesis, the development of cell walls, and the formation of fruit color [47].

Anthocyanins, secondary metabolites found in fruit and vegetables, serve multiple functions. They play an important role in regulating photosynthesis, filtering UV rays, and greatly boosting the antioxidant capacity of fruit and vegetables. Research findings indicate that LNC1 and LNC2 function as endogenous target mimics (eTMs) for miR156a and miR828a, respectively, thereby regulating the expression of TFs (SQUAMOSA promoter-binding protein-like 9) *SPL9* and *MYB114*. This regulatory mechanism impacts the anthocyanin content in sea buckthorn fruit [21]. In mulberry, lncNAT (ABCB19AS), derived from ABC transporter B19 (ABCB19), induces cleavage by miR477 to promote anthocyanin accumulation by regulating *ABCB19* expression [48]. In apple, a transcriptional cascade involving WRKY1–LNC499–ERF109 has been identified. WRKY1 activates the transcription of *LNC499* by specifically binding to the W-box on the LNC499 promoter, leading to the upregulation of *ERF109*. The ERF109 protein induces the expression of genes related to anthocyanin production during the early stage of apple coloring, thereby promoting anthocyanin accumulation. This entire transcriptional cascade regulates the anthocyanin content in apple fruit [49]. Moreover, MLNC3.2 and MLNC4.6 function as eTMs of miR156, inhibiting the cleavage of SPL2-like and SPL33 transcripts by miR156a during photoinduced anthocyanin biosynthesis in apple. This involvement allows them to participate in the anthocyanin synthesis pathway [24]. Additionally, the expression of *LNC610* [50] and *LINC15957* [51] has also been reported to enhance anthocyanin accumulation in apple and radish, respectively.

Carotenoids are crucial pigments found in plant leaves, flowers, and fruit. They attract pollen and seed dispersers for pollination and seed dispersal while also providing protection against damage from bright light. In two differently colored mature sea buckthorn fruits, 61 DELs have been identified, with 23 specifically expressed in red fruit and 22 in yellow fruit. These DELs play a role in carotenoid biosynthesis by regulating target genes in either *cis* or *trans* mechanisms [22]. Similarly, in *Capsicum annuum*, 2505 lncRNAs were identified, with 1066 differentially expressed during fruit development. Numerous potential PGs targeted by these DELs with *cis* or *trans* action participate in carotenoid biosynthesis [16].

#### Reproductive tissue development

Flowering marks the beginning of the reproductive phase in plants, wherein the meristem located at the apex of the stem undergoes a transformation, giving rise to anthers, pollen, and other inflorescence meristems. The development of reproductive tissue in fruit and vegetables is crucial for their reproduction and is regulated by various factors. Extensive research has been conducted on the role of lncRNAs in the reproductive tissues of fruit and vegetables. Sequencing of *Solanum lycopersicum* Heinz 1706 and *Solanum pimpinellifolium* LA1589 tomatoes has identified tissue-specific lncRNAs, with 62% in Heinz 1706 and 44% in LA1589 specifically expressed in reproductive tissues [52]. In addition, Yang *et al*. [53] identified a total of 10 919 lncRNAs in the leaves, flowers, and roots of tomatoes, which play vital roles in regulating the formation of the flower intima. In cucumber, 3274 lncRNAs associated with sexual differentiation and fruit development were identified, with 94 of them found to play a role in reproductive and sexual differentiation processes [54]. In mulberry, 1133 lncRNAs were identified in various tissues, with 106 of them exhibiting tissue-specific expression. Among these, LNC_0132, LNC_0521, and LNC_0782 are located near coding genes involved in flower development and exhibit specific expression in flowers [55]. The formation of multiple pistils in *Prunus mume* is associated with two lncRNAs, XR_514690.2 and TCON_00032517, and a total of 2572 lncRNAs involved in flower development were identified [56]. Furthermore, lncRNAs associated with both pollen and flower development have also been identified in other horticultural crops, such as *Brassica rapa* [57], *F. vesca* [42], and *Poncirus trifoliata* [58]. It is worth noting that some fruit and vegetables require a period of sustained low temperature, known as vernalization, to facilitate the emergence of reproductive buds and the transition from vegetative growth to reproductive growth. In *Beta vulgaris*, three lncRNAs (GL15X1, AGL15X2, and CAULIFLOWER A) have been found to be associated with the vernalization process [59]. Additionally, in *Brassica campestris*, the lncRNA BcMF1, which is specific to pollen, plays a critical role in ensuring efficient pollen germination and pollen tube elongation. Suppression of *BcMF1* expression results in various abnormal phenotypes during pollen development, such as delayed degradation of the tapetal layer, and extensive pollen grain atrophy [60].

#### Fruit ripening

Fruit ripening is a genetically regulated, highly coordinated, and irreversible process that represents a distinct stage in the life cycle of higher plants [61]. It is characterized by a series of physiological, biochemical, and sensory changes in the fruit, ultimately leading to optimal fruit quality. In recent years, numerous lncRNAs have been identified to participate in the ripening process across various fruits, including tomato [32], kiwifruit [62], sea buckthorn [21], melon [63], and peach [26]. In apple, specific lncRNAs expressed during the early stages of fruit development and maturation play a crucial role. They are involved in a multitude of biological processes, including energy production and transformation, and carbohydrate transport and metabolism, as well as post-translational modification and protein conversion [64]. Similarly, in melon, multiple lncRNAs with high expression abundance have been identified at different maturation stages. Enrichment analysis has revealed their involvement in fruit growth, development, and ripening through the mediation of auxin signal transduction, ethylene (ET) and sucrose biosynthesis and metabolism, the abscisic acid (ABA) signaling pathway, and TF regulation [63]. Furthermore, significant progress has been made in unraveling the roles and regulatory mechanisms of certain lncRNAs in the intricate process of fruit ripening. For instance, miR397 has been identified as a key regulator of strawberry ripening, acting by cleaving lincRNA FRILAIR transcripts associated with fruit ripening. Notably, overexpression of *FRILAIR* in strawberry fruit leads to an accelerated ripening phenotype [9]. In addition, the loss of function of several lncRNAs, including lncRNA1459 [65], lncRNA1840 [32], and lncRNA2155 [66], has been found to inhibit ET production and lycopene accumulation, leading to a ripening inhibition phenotype in tomato fruit. Moreover, the expression of *lncRNA314* was found to be significantly upregulated during the breaker and ripening stages of tomato fruit, and correlation analysis has revealed its co-expression with the adjacent ATP binding cassette (ABC) transporter gene. Interestingly, its expression is restricted in a tomato ripening mutant [52]. In apple, three lncRNAs (TCON_00131720, TCON_00025525, and TCON_00032426) have been identified as targets associated with auxin/indole-3-acetic acid 32, SAUR-like auxin-responsive protein (SAUR36), and peroxidase A2-like, respectively. These lncRNAs are implicated in the regulation of apple fruit ripening [25]. Additionally, in the context of grape berry ripening, six lncRNAs (TCONS 00221683, TCONS 00684459, TCONS 00022149, TCONS 00167247, TCONS 00258125, and TCONS 00261813) have been pinpointed as key regulators [35]. It is worth noting that fruit aging is closely linked to fruit quality and stress response, resulting in significant changes in fruit color, texture, flavor, nutritional value, and resistance. In pear fruit, a total of 3330 lncRNAs have been identified with 2060 and 537 lncRNAs responsive to high- and low-temperature conditions, respectively. Among these DELs, 82 and 24 have been associated with fruit senescence, and 33 lncRNAs have been predicted to be involved in fruit senescence regulation through the competing endogenous RNA (ceRNA) network under varying temperature and pressure conditions. Moreover, the interaction between LNC_000249-miR172, LNC_000862-miR390a, and LNC_002622-Novel_173 modulates the expression of *pbro25174.1*, *pbr031098.1*, and *pbro18118.1*, respectively, thereby regulating the accumulation of anti-aging compounds [28]. This demonstrates the intricate web of lncRNA-mediated regulation in fruit ripening and aging.

#### Other metabolic processes

Granulation, a post-harvest disorder in navel orange fruit, is characterized by the spread of granulation from the fruit stem, leading to reduced sugar and organic acid content in the juice sac and sensory deterioration. In orange, Yao *et al*. [39] identified 486 lncRNAs involved in orange granulation through the regulation of genes associated with cell wall metabolism and cellulose biosynthesis, metabolism, and enzyme activity. Further analysis of the differentially expressed genes (DEGs) during granulation revealed their potential role in granulation through the regulation of cell wall metabolism-associated genes. Bud endogenous dormancy, a response to cyclical environmental changes, relies on gene expression regulation influenced by low temperature and a short photoperiod for bud release. Failure to break endodormancy results in unsuccessful flowering. In *Pyrus pyrifolia*, Pp-miRn182, derived from lncRNA PpL-T31511, targets the type 2C protein phosphatase 1 (PP2C1) and participates in hydrogen cyanide-induced endodormancy release via the PP2C–H_2_O_2_ pathway [67]. Browning is a common phenomenon in fruit and vegetables during post-harvest storage and fresh cutting, resulting in appearance loss, as well as reduced storage duration, flavor, and nutritional value. Through SMRT-seq and RNA-seq analyses of two browning varieties, a total of 254 lncRNAs were identified. Notably, specific lncRNAs, such as PB.15038, PB.156.1, PB.7534.1, and PB.8714.2, were found to target coding genes associated with peroxidase (POD), polyphenol oxidase (PPO), chalcone synthase (CHS), and cinnamoyl-CoA reductase (CCR), respectively. Additionally, lncRNA PB.6461.1 and PB.13941.1 target 4-coumarate:CoA ligase (4CL), which regulates enzymatic browning in sand pear flesh by targeting relevant enzyme-coding genes [68]. Secondary metabolites significantly contribute to the market value and overall quality of fruit and vegetables, including vitamin C, citric acid, flavonoids, and stress-responsive terpenoids. In four lemon tissues, 11 814 lncRNAs were identified, with 632 lncRNAs showing a high correlation with 5810 mRNAs. Among them, 113 lncRNAs were involved in terpenoid metabolism through associated mRNAs, while 29 lncRNAs played a role in flavonoid metabolism pathways [19]. In the three ripening stages of the peach fruit, a total of 575 DELs were identified, and enrichment analysis indicated their potential contribution to the physiological and metabolic changes associated with fruit ripening, particularly in flavonoid biosynthesis and aroma compound accumulation [26].

### Role of lncRNAs in the stress response of fruit and vegetables

#### Biotic stress

Crops are susceptible to various biological stresses both before and after harvest, which significantly impact their growth, development, and overall quality. These stresses include pathogens, insect pests, and parasitic organisms, resulting in considerable losses in fruit and vegetable yield and quality. Pathogen infections are a major cause of fruit and vegetable diseases, contributing significantly to spoilage. In response to pathogen infections, fruit and vegetables undergo a dynamic molecular response to enhance their immune capabilities. For instance, in tomato plants a study identified 196 lncRNAs that respond to *Phytophthora infestans* infection. Among them, 148 lncRNAs regulate the expression of 771 genes through 887 lncRNA–mRNA pairs, participating in the resistance reaction. Functional analysis demonstrated that lncRNA42705 and lncRNA08700, acting as targets of miR159, influence the expression of MYB and enhance tomato resistance to *P. infestans* [69]. Additionally, lncRNA23468 and lncRNA08489 function as eTM regulatory nucleotide-binding site–leucine-rich repeats (NBS-LRRs) for miR482b and miR482e-3p, respectively. The expression of *NBS-LRR* is involved in tomato resistance to *P. infestans* through the ROS clearance system. Another lncRNA, lncRNA16397, which is an antisense transcript of the glutaredoxin gene (*GRX22*), induces *GRX21* expression, reduces ROS accumulation, alleviates cell membrane damage, and enhances resistance to *P. infestans* [70]. Furthermore, the expression of *lncRNA33732* induces the activity of respiratory burst oxidase (RBOH), leading to the accumulation of H_2_O_2_ and enhancing tomato resistance to *P. infestans* [10]. Wang *et al*. [71]identified 2056 lncRNAs (including 1767 lincRNAs and 289 lncNATs) in response to tomato yellow leaf curl virus (TYLCV) infection and confirmed the positive role of *LNC0957* expression in tomato resistance to TYLCV. Additionally, LNC1077 and LNC0195 participate in the response of tomatoes to TYLCV infection as targets of miR399 and miR166, respectively [72]. Normal transcription of *LNR1* contributes to tomato resistance to TYLCV, while overexpression inhibits TYLCV accumulation, and silencing LNR1 leads to a phenotype of TYLCV infection in tomato [73]. Zhou *et al*. [74]reported that *Bacillus subtilis* SL18r triggers tomato resistance to *Botrytis cinerea* by activating lncRNA MSTRG18363, which functions as a decoy to suppress miR1918, resulting in the inhibition of its target gene *SlATL20*, ultimately inducing systemic pathogen resistance. In various melon varieties, 407 and 611 DELs are involved in powdery mildew disease infection, and their regulation occurs through stress response targeting [75]. In *Brassica napus*, 931 lncRNAs were identified as responsive to *Sclerotinia sclerotiorum* infection [76]. Furthermore, 464 lncRNAs exhibited differential expression in clubroot-sensitive lines of rapeseed. Enrichment analysis revealed that these lncRNAs participate in clubroot resistance through interactions with pathogens, hormone signaling pathways, and primary and secondary metabolic pathways [77].

#### Abiotic stress

##### Fruit cracking

Fruit cracking is a serious physiological disorder that not only renders fruit susceptible to pathogens and water loss but also compromises its postharvest storage quality. Fruit cracking is mostly caused by the disharmony between the storage environment and internal growth of the fruit, as well as the rupture of the fruit epidermis or cuticle, and, more seriously, it affects the pulp tissue, providing an entry point for diseases and pests, thereby shortening the fruit’s storage life. In-depth sequencing analysis of tomato fruit, both resistant and susceptible to cracking, identified 21 048 lncRNA–mRNA targeting relationships. Analysis of the lncRNA–mRNA regulatory network revealed that specific lncRNAs coordinate the expression of related genes in the hormone–redox cell wall module, thereby regulating tomato fruit cracking. Notably, lncRNAs such as XLOC_033910, XLOC_007053, and XLOC_008464 were found to play crucial roles in this regulation [38]. Bagging is an effective measure to prevent fruit cracking in pomegranate. Analysis of lncRNAs libraries from cracked, uncracked, and bagged pomegranate fruits identified 3194 lncRNAs, with 42 and 35 DELs identified in the cracked versus uncracked fruit group and cracked versus non-cracked fruit after bagging group, respectively. These DELs are involved in calcium ion binding, glycerophospholipid metabolism, flavonoid biosynthesis, cell wall biogenesis, xylodextran metabolism, hormone signaling, and starch and sucrose metabolism through *cis*- or *trans*-regulated differential transcripts, thus influencing the cracking of pomegranate fruit [27].

##### Nutritional stress

Phosphorus (Pi) is an essential nutrient for the growth and development of fruit and vegetables. However, the utilization efficiency of Pi in fruit and vegetables crops is very low, and the unabsorbed Pi will cause environmental pollution. Therefore, studying the molecular regulatory mechanisms of fruit and vegetables under Pi starvation conditions is of great significance to ensure their growth, development, and yield. In tomato, a Pi starvation-induced lncRNA TPSI1 has been identified at an early stage. TPSI1 shows rapid upregulation in roots and leaves under Pi starvation, followed by a rapid decrease after Pi supplementation [78]. This suggests lncRNA involvement in the early response to Pi starvation. In addition, iron (Fe) is another essential nutrient for plant growth. In apple, it has been found that Fe deficiency induces the expression of lncRNA *MSTRG.85814* in the roots, specifically its splicing variant *MSTRG.85814.11*, which targets the mRNA of SAUR32 and activates proton extrusion in response to Fe deficiency [79].

##### Temperature stress

Low-temperature storage is an effective method to delay the metabolic process of fruit and vegetables, preserving their quality. However, some cold-sensitive produce, such as tomato, banana, and mango, are susceptible to freezing damage at low temperatures, resulting in a significant decline in their quality. In tomato, 239 DELs were identified in response to low-temperature stress, and functional analysis showed that these DELs mediated the tomato fruit’s response to cold stress by influencing the expression of enzymes related to redox reactions, cell wall degradation, membrane lipid catalase, cold and heat shock proteins, energy metabolism, and salicylic acid (SA) and ABA metabolism [29]. In mango, a total of 7610 lncRNAs were identified in response to temperature changes. Notably, lnc26299 was found to have the capability to interact with related cDNA 12B (RC12B), a protein that exhibited significant upregulation in response to cold stress [80]. Additionally, Lai *et al*. [62] found that lncRNAs play an important regulatory role in the low-temperature storage of kiwifruit by mediating the expression of genes related to starch and sucrose metabolism and cell wall modification. High-temperature stress can disrupt cellular homeostasis, hinder the growth of fruit and vegetables, and reduce their stress resistance. In cucumber, a total of 2085 lncRNAs have been identified in the response to high-temperature stress. Among these, TCON_00031790, TCON_00014332, TCON_00014717, and TCON_00005674 interact with miR9748 through the plant hormone signal transduction pathway in response to high-temperature stress [81]. Furthermore, 10 001 lncRNAs in headless Chinese cabbage were identified as responsive to temperature changes [82].

##### Salt stress

Salinity is a crucial environmental factor that limits plant growth and development. High salinity exerts detrimental effects on crop productivity, impacting various physiological and biochemical processes. To investigate the molecular mechanisms underlying tomato salt tolerance, a high-throughput sequencing analysis was conducted on both wild-type and cultivated tomatoes with high salt tolerance. In tomato, the functions of salt-induced lncRNAs were reported by Li *et al*. [33], who found that the target genes of these lncRNAs were closely related to some pathways, such as phytohormone metabolism, photosynthesis, and protein/amino acid metabolism. These lncRNAs might respond to the salt stress process by interacting with microRNAs (miRNAs), and these interactions vary among different tomato varieties depending on their salt stress resistance levels. In addition, Li *et al*. [83] found that miRNA–lncRNA–mRNA networks play important roles in regulating gene expression to modify growth, improve photosynthesis, glycometabolism, and energy metabolism, adjust plasma membrane permeability, regulate TF, and participate in the phosphoinositol signaling system during adaptation to salt stress in sugar beet. In salt-induced grape roots, a total of 1661 DELs were identified, which regulated the expression of 546, 771, and 608 mRNAs through *cis*-, *trans*- and miRNA-mediated mechanisms, respectively. These DELs were involved in transcriptional regulation, ubiquitin–proteasome pathways, multi-heavy ion binding, and electron carrier activity [84].

##### Drought stress

Drought is a significant abiotic stress that affects global crop yield. It impacts various aspects of plant biology, including male organ development, stomatal movement, morphological changes, biosynthetic and antioxidant pathways, and respiratory pathways. Some lncRNAs have been implicated in plant responses to drought stress. For instance, the TF CYCLING DOF FACTOR 1 (StCDF1), a central regulator of the circadian clock, has a natural antisense transcript (StFLORE) with antiphasic gene expression over the circadian cycle. StFLORE regulates water loss by affecting stomatal growth and diurnal opening in *Solanum tuberosum* [85]. In soybeans, an abiotic stress-related lncRNA, namely lncRNA77580, was identified, and its overexpression enhanced drought tolerance [86]. Drought-responsive lncRNAs have also been identified in various fruits and vegetables, including sugar beet [87], tomato [88], *Brassica juncea* [89], and *B. napus* [90]. To unravel the regulatory mechanisms of lncRNAs in tomato under drought stress, Eom *et al*. [91] conducted a comprehensive analysis of transcriptome data from drought-treated tomato leaves, and identified 521 drought-responsive lncRNAs, which were found to target 92 miRNAs and 183 mRNAs, thereby regulating stimulus response and signal transduction pathways. Notably, the drought-induced lncRNA467 was found to potentially impact stomatal motility by targeting *Solyc11g011500*, while lncRNA025 was observed to enhance chloroplast energy balance in response to drought stress.

## Molecular function of lncRNAs

### LncRNAs and gene expression

**Figure 3:**
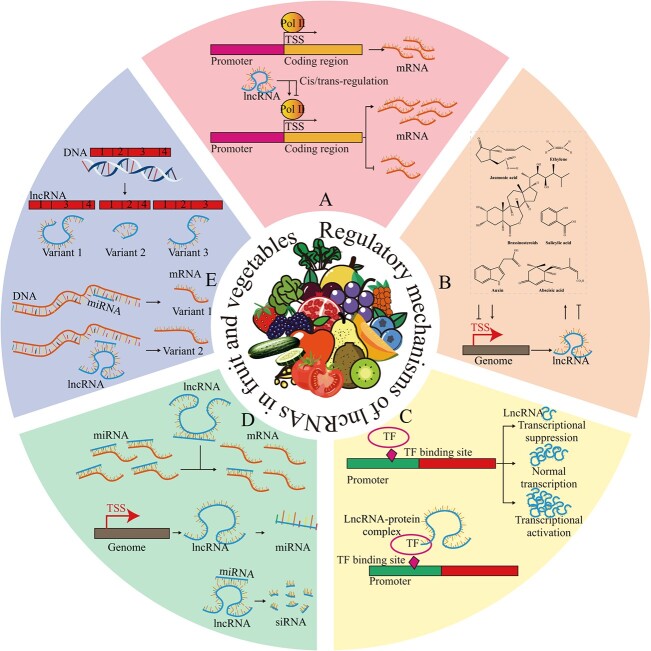
Regulatory mechanisms of lncRNAs in fruit and vegetables. **A** lncRNAs regulate the expression of genes in *cis* or *trans* configuration. **B** Mutual regulation of lncRNAs and plant hormones. **C** TFs activate or inhibit the expression of lncRNAs by recognizing transcription recognition sites within the lncRNA promoter region; lncRNAs bound to transcription factors, forming RNA–protein complexes that modulate the activity of transcription factors. **D** In the first scenario, lncRNAs act as a precursor for miRNA. In the second scenario, miRNAs recognize and cleave lncRNAs to generate siRNA; lncRNAs are competitively bound to miRNAs, regulating the expression of target mRNAs. **E** Alternative splicing generates diverse variants of lncRNAs; lncRNAs bind to miRNAs, altering the regulatory function of miRNAs on mRNAs and leading to the production of distinct mRNA variants.

Genes serve as the fundamental units governing the genetic characteristics of living organisms. The regulation of gene expression, including transcriptional control and protein translation modification, is crucial for the viability and phenotypic alterations of plants. Extensive research has been conducted into the relationship between lncRNAs and gene expression. For example, the knockout of lncRNA1459 in tomato fruit resulted in a large number of DEGs and DELs, including genes involved in ET and carotenoid biosynthesis, which exhibited significant downregulation [65]. lncRNAs regulate gene expression primarily through *cis*/*trans* mechanisms (Fig. 3A). The *cis*-regulatory effects of lncRNAs can be classified into three types: (i) the lncRNA transcript itself modulates the expression of adjacent genes by its ability to recruit regulatory factors to loci and/or regulate their function; (ii) the transcription and/or splicing of the lncRNAs itself can confer gene-regulatory functions that are independent of the RNA transcript’s sequence; and (iii) the *cis*-regulation is solely determined by DNA elements within the lncRNA promoter or gene locus and is completely independent of the coding RNA or its production. The *trans*-regulatory effects of lncRNAs can also be classified into three types: (i) lncRNAs that regulate chromatin states and gene expression in regions distant from their transcription site; (ii) lncRNAs that influence nuclear structure and organization; and (iii) lncRNAs that interact with and regulate the behavior of proteins and/or other RNA molecules. By employing predictive techniques to evaluate the correlation in mRNA expression between lncRNAs and their adjacent genes within the 100-kb region both upstream and downstream, it becomes feasible to effectively analyze the *cis*-regulatory target gene pairs associated with the lncRNAs. Furthermore, identifying lncRNA *trans*-regulatory relationships requires a larger sample size (exceeding six samples), and co-expression analysis can predict *trans*-regulatory target gene pairs that are not in close proximity to the lncRNAs (beyond the 100-kb region) [38]. A large number of lncRNAs have been identified in fruit and vegetables that can regulate mRNA expression in a *cis*/*trans* manner. For example, in *Prunus persica*, Zhou *et al*. [20] identified 575 lncRNAs that regulate the expression of 7103 mRNAs through *cis*/*trans* mechanisms, with an average of 12 target mRNAs per lncRNA. In apples, a novel lncRNA, lncRNA_PG1_, was shown to be located in the promoter region of *polygalacturonase 1* (*MdPG1*) and inhibited the expression of *MdPG1* by *cis* action [92]. In a study by Zhang *et al*. [22] on sea buckthorn fruit, a total of 2303 lncRNAs were identified as regulators of gene expression in *cis*, while 2762 lncRNAs were found to regulate gene expression in *trans*. Notably, certain lncRNAs, such as XLOC_267510, XLOC_338163, and XLOC_169881, exhibited both *cis* and *trans* regulatory roles. Similar regulatory mechanisms of lncRNAs in gene expression have also been reported in many other fruits and vegetables, including tomato [30], hot pepper [16], and *Cucumis melo* [63]. In recent years, numerous reports have highlighted the impact of lncRNAs on fruit and vegetable quality through gene expression regulation. However, comprehensive studies on the mechanism of lncRNA-mediated gene expression regulation, specifically in fruit and vegetables, are lacking. Particularly, the influence of lncRNAs on chromatin modification and nuclear modification in fruit and vegetables remains unexplored. However, further experimental evidence is required to demonstrate the *cis*/*trans* regulatory mechanisms of lncRNAs in fruit and vegetables.

### lncRNAs and plant hormones

Plant hormones, including cytokinin, ABA, ET, brassinosteroid (BR), jasmonic acid (JA), and SA, play a crucial role in coordinating various life activities, such as the growth and development of fruit and vegetable crops. These trace endogenous substances are involved in intricate molecular regulatory networks, regulating gene expression, enzyme activity reactions, and signal cascade reactions, which also include the regulation of lncRNAs (Fig. 3B). For example, 382 and 78 lncRNAs were identified in response to ABA treatment in strawberries [45]. In kiwifruit, 78 lncRNAs were identified in response to ABA treatment, and the upregulation of these lncRNAs induced by ABA treatment led to increased ET biosynthesis and fruit softening [13]. A new lncRNA, TCON _00202033, has been found to be associated with SA-mediated signaling pathways, playing a role in the innate immune response of kiwifruit [15]. Similarly, lncRNAs also play a crucial regulatory role in the synthesis and signal transduction of plant hormones, thereby influencing the development, ripening, and stress responses of fruit and vegetables. In tomato fruit, numerous lncRNAs have been found to target genes associated with ET signaling, such as auxin response factor, induction protein, F-box proteins, ERFs, and MADS-box proteins [93]. Additionally, some lncRNAs can modulate the response to salt stress by controlling the synthesis and signaling pathways of ABA, BR, and ET (Fig. 3B) [33]. In our previous study, we found that lncRNA4504 mediates methyl jasmonate-induced fruit resistance to gray mold by upregulating the expression of genes associated with the JA signaling pathway [94]. Additionally, in *C. melo*, LNC_002345 and LNC_000154 were found to potentially co-regulate with multiple genes involved in auxin signal transduction and act in the same pathways, while lncRNAs associated with fruit ripening and the climacteric phase (LNC_000987, LNC_000693, LNC_001323, LNC_003610, LNC_001263, and LNC_003380) may participate in the regulation of ET biosynthesis and metabolism, as well as the ABA signaling pathway [63]. In *P. mume*, the lncRNA TCON_00032517 might contribute to the formation of multiple pistils by inducing the expression of the cytokinin negative regulator gene A-ARR [56]. In pear fruit, the lncRNA PpL-T31511 is involved in the regulation of bud dormancy by influencing the expression of *2C protein phosphatase* (*PP2C*), a crucial component in the ABA signaling pathway [67]. In mango, Moh *et al*. [80] identified a heat-responsive lncRNA, HRlnc11351, which plays a role in mango development and stress response by targeting 3-ketoacyl-CoA thiolase 2, an enzyme involved in the β-oxidation of fatty acids as well as ABA signal transduction. The above findings indicate that the interaction between plant hormones and lncRNAs establishes a versatile regulatory mechanism that impacts the development and physiology of fruit and vegetables. It is important to note that most of these regulatory associations between lncRNAs and plant hormones have been inferred through omics analysis combined with bioinformatics predictions. Therefore, further investigation is necessary to gain a comprehensive understanding of the precise roles and regulatory mechanisms of lncRNAs in the biosynthesis and signal transduction pathways of various plant hormones.

### LncRNAs and transcription factors

TFs, similar to plant hormones, play a crucial role in regulating various life processes, including the development, ripening, and stress responses of fruit and vegetables. TFs recognize promoter elements to orchestrate gene expression at the transcriptional level. Notably, similar to PG, the transcription of lncRNAs also relies on the recognition of their promoters by TFs (Fig. 3C). For example, Yu *et al*. [66] identified 187 lncRNAs that were directly targeted by TF ripening inhibitor (RIN), with each promoter containing RIN binding sites. Cui *et al*. [10] also discovered 199 lncRNAs that significantly contribute to WRKY1-mediated resistance to *P. infestans*, with 22 of their promoter regions containing WRKY1 recognition sites. Among these, lncRNA33732 was activated by WRKY1 through specific interactions with the W-box element in its promoter, leading to the induction of respiratory burst oxidase expression and an increase in H_2_O_2_ accumulation in the early defense reaction of tomato to *P. infestans* attack. On the other hand, lncRNAs can also regulate the activities and functions of TFs through various mechanisms, thereby controlling the metabolic processes of fruit and vegetables (Fig. 3C). For example, in tomato fruit, lncRNAZ078 targets ERFs, while lncRNAZ107 and lncRNAZ141 target MADS-box proteins and F-box proteins, respectively, to participate in regulating ET metabolism [93]. Overexpressing *LINC15957* in *Raphanus sativus* leads to distinct expression patterns of several TFs, including MYB, bHLH, WD40, bZIP, ERF, WRKY, and MATE [51]. In apple, WRKY1 activates the expression of *LNC499* by targeting its promoter, which subsequently regulates the expression of *ERF109* through *cis*-regulation [95]. In different development stages of grape, a total of 56 441 lncRNAs were identified, and these lncRNAs were found to interact with 19 TF families, including AP2, ERF, bHLH, bZIP, C3HL, and ERF, collectively contributing to the regulation of fruit development. Additionally, alterations in lncRNA expression can also indirectly influence the activity of TFs through mechanisms such as miRNA regulation and other targets. For example, in tomato, several lncRNAs, namely lncRNA42705/lncRNA08711, lncRNA39896, and lncRNA11265/lncRNA15816, have the potential to function as eTMs for miR159, miR166b, and miR164a-5p, respectively. This interaction modulates the activities of MYB, HD-Zip, and NAC TFs, consequently playing a role in tomato resistance to *P. infestans* [69]. In *P. mume*, the lncRNA TCON 00032517 modulates the expression of AP2 TF through the regulation of its target gene, significantly influencing flower development [56]. Based on the above findings, it is clear that the interaction between lncRNAs and TFs is heavily involved in the regulation of various aspects of fruit and vegetable life processes.

### lncRNAs and microRNA

Numerous studies have demonstrated that lncRNAs are involved in the physiological processes and stress responses of fruit and vegetables via interactions with miRNAs. miRNAs are short ncRNAs consisting of 2124 nt that play crucial roles in post-transcriptional regulation by inhibiting gene translation or degrading target mRNAs. Through the regulation of miRNAs, lncRNAs play a role in the metabolic regulation of fruit and vegetables. Due to the presence of eTMs of mature miRNAs within lncRNAs, lncRNAs act as ceRNAs to decoy mature miRNAs and thereby suppress their expression (Fig. 3D). For example, tomato lncRNA39026, lncRNA23468, and lncRNA08489 contain the eTMs of mature miR168a, miRNA482b, and miR482-3p, respectively, to inhibit their expression, increasing the immunity of tomato to *P. infestans*. In apple, two putative eTMs (MLNC3.2 and MLNC4.6) have been identified for miRNA156a. These lncRNAs inhibit miR156a from cleaving squamosa promoter-binding protein-like (SPL) TFs SPL2-like and SPL3, which in turn control anthocyanin biosynthesis under photoinduced conditions [24]. Different from the mechanism mentioned above, certain lncRNAs, such as tomato lncRNA15492, can inhibit the expression of mature miR482a by binding with pre-miR482a, which is located on the antisense sequence of lncRNA15492 [96]. Additionally, some lncRNAs containing pre-miRNA sequences can act as miRNA precursors, thereby promoting miRNA expression (Fig. 3D). Currently, omics analysis has identified multiple lncRNAs that can act as miRNA precursors and promote the production of miRNAs in many horticultural crops, such as rape [76], melon [75], and tomato [30], contributing to their development and stress responses. On the other hand, the expression and function of lncRNAs can be regulated through miRNA cleavage. Yang *et al*. [73] found that a specific lncRNA named SlLNR is cleaved by small interfering RNAs (siRNAs) from TYLCV, resulting in the inhibition of its expression. Additionally, the cleavage of lncRNAs by miRNAs can trigger the generation of phased, secondary, siRNA (phasiRNA) (Fig. 3D). For example, 25-nt viral small-interfering RNAs derived from non-coding intergenic regions of tomato can disrupt SlLNR1, leading to its silencing and rendering tomato plants more susceptible to TYLCV infection. In mulberry, Gai *et al*. [97] revealed that miR3954 targets LNC1, resulting in the production of a 21-nt siRNA (si161579). This siRNA regulates the expression of *calmodulin-like 27* (*MuCML27*), conferring resistance to pathogens as well as contributing to salt and drought stress responses. Additionally, in tomato, a particular interaction model between lncRNA15492 and miR482a was discovered by Jiang *et al*. [96], who proposed that lncRNA15492 could inhibit the expression of mature miR482a because pre-miR482a is located on the antisense sequence of lncRNA15492, while mature miR482a could also cleave lncRNA15492 to relieve the inhibition of pre-miR482a, leading to an increase in the accumulation of mature miR482a. This interaction between miR482a and lncRNA15492 affects the resistance of tomatoes to *P. infestans* by maintaining the homeostasis of NBS-LRR. The above research results indicate that lncRNAs can play important regulatory roles in the development and stress response of fruit and vegetables through various interactions with miRNAs.

### lncRNAs and alternative splicing

Alternative splicing (AS) is prevalent in eukaryotic plants and plays a crucial role in the complexity of the biological transcriptome and proteome by modulating gene splicing sites, which significantly contributes to the growth and development of fruit and vegetables [98]. The diversity of lncRNAs is intrinsically linked to AS, as different modes of AS can generate multiple lncRNA variants from the same genomic region (Fig. 3E). For instance, in pear, a single gene can produce four lncRNA variants (LNC_000443, LNC_000444, LNC_000445, and LNC_000446) through distinct AS modes [28]. Similarly, during the early flowering stage in tomatoes, 16 995 AS events were identified in 72.55% of lncRNAs across flowers, leaves, and roots [98]. Additionally, lncRNAs can regulate gene expression by interacting with AS factors, impacting their function. They can also form double-stranded complements with pre-mRNA, thereby affecting the splicing of targeted pre-mRNA molecules (Fig. 3E). Moreover, lncRNAs can affect target gene chromatin remodeling, which indirectly affects pre-mRNA AS. A comprehensive understanding of the regulatory interplay between lncRNAs and AS can optimize their relationship, ultimately leading to improved fruit and vegetable quality. As an example, the *trans*-splicing interaction between ACoS-AS1 and the PSY coding gene *PSY1* affects the functionality of *PSY1*, leading to the yellow phenotype in tomato fruit. Conversely, CRISPR/Cas9 knockout of ACoS-AS1 results in a red phenotype in tomato fruit [8].

## Conclusions and perspective

The development of high-throughput sequencing technology has greatly enhanced the ability to discover, predict, and identify lncRNAs. Techniques such as paired-end strand-specific RNA sequencing have continuously revealed a growing number of lncRNAs expressed in fruit and vegetable crops [13, 19, 28]. However, there is an absence of substantial lncRNA data in plant databases specifically related to fruit and vegetables, such as LncPheDB, CANTATAdb, and PlncRNADB, which predominantly focus on food crops. Currently, there is an urgent need to develop a comprehensive collection of lncRNAs associated with fruit and vegetables. This collection should resemble the human lncRNA database, LncSEA, and facilitate the annotation and concentration analysis of lncRNAs for the purpose of analyzing their regulatory effects both upstream and downstream [99]. In terms of lncRNA localization, tools like LncLocator, iLoc-LncRNA, and RNALocate have been developed to predict lncRNA subcellular localization. Traditional methods for lncRNA localization verification, such as FISH, are rarely used in fruit and vegetable analysis due to the particularities (such as plant autofluorescence) of plant tissues and the limited applicability of FISH technology. Instead, the karyoplasmic localization information of lncRNAs is often determined through the separation of nuclear and cytoplasmic RNA in fruit and vegetable tissues. Subsequently, the regulatory relationship between lncRNAs and miRNAs is further analyzed. Functional exploration of lncRNAs in fruit and vegetable crops can be achieved by overexpression, RNAi, and CRISPR/Cas9 genome editing techniques. However, the limitations of these technologies confront uncertainties in the functional analysis and mechanism exploration of fruit and vegetable lncRNAs. Despite the development of prediction methods for exploring the regulatory mechanisms of lncRNAs in fruit and vegetables, such as CNN, IndRNN, PmliPred, DRPLPI, and PLncWX, the confidence and applicability of the prediction results are restricted by database limitations. Despite the fact that our paper demonstrates that lncRNAs play a role in a variety of biological processes in fruit and vegetable crops, such as pigment accumulation, reproductive tissue development, fruit ripening, and stress responses, research on lncRNAs in fruit and vegetable crops is still in its early phase. In particular, little research has been done on the precise regulatory mechanisms of lncRNAs in fruit and vegetable crops. Research methods for lncRNAs in animals and even in model plants like *Arabidopsis thaliana* should be extended to lncRNAs in fruit and vegetable crops. The incomplete biological information database of fruit and vegetable lncRNAs, especially the lack of gene chip data, seriously affects the function prediction and regulatory mechanism studies of lncRNAs. The exploration of the function and mechanism of lncRNAs in fruit and vegetables crops has just begun, and rapid progress and development of technology will bring new opportunities and breakthroughs for lncRNA research in fruit and vegetable crops.
